# Association Between Obesity Severity and Peripartum Maternal and Neonatal Outcomes Among Advanced Maternal Age Pregnant Women

**DOI:** 10.7759/cureus.112704

**Published:** 2026-07-15

**Authors:** Turki F Alqumei, Salman M Alosaimi, Layan M Alqedheeb, Faisal S Alhafi, Mohammed A Alqout, Nada A Alsaigh

**Affiliations:** 1 College of Medicine, King Saud bin Abdulaziz University for Health Sciences, Riyadh, SAU; 2 College of Medicine, Prince Sattam bin Abdulaziz University, Al-Kharj, SAU; 3 College of Medicine, Qassim University, Buraidah, SAU; 4 College of Medicine, King Saud University, Riyadh, SAU; 5 Obstetrics and Gynaecology, King Abdulaziz Medical City, Riyadh, SAU

**Keywords:** advanced maternal age pregnancy, birth weight, caesarian delivery, labor and delivery complications, maternal and neonatal complications, maternal obesity

## Abstract

Background

Obesity and advanced maternal age are both associated with adverse maternal and neonatal outcomes. However, the effect of increasing obesity severity within an advanced maternal age population remains insufficiently characterized, particularly in regional cohorts.

Objective

The objective of this study is to investigate the association between obesity severity and peripartum maternal and neonatal outcomes among pregnant women of advanced maternal age.

Methods

This retrospective cohort study was conducted at the Specialized Hospital for Women’s Health, Ministry of National Guard Health Affairs, Riyadh, Saudi Arabia. Medical records of pregnant women aged 35 years or older with obesity who delivered between 2022 and 2025 were reviewed using the BESTCare database. Women were classified into obesity Class I, Class II, and Class III according to body mass index. Maternal, obstetric, and neonatal outcomes were compared across obesity classes using appropriate statistical tests.

Results

A total of 1162 women were included, of whom 478 (41.1%) had Class I obesity, 424 (36.5%) had Class II obesity, and 260 (22.4%) had Class III obesity. Mode of delivery differed significantly across obesity classes (P=.017), with the highest cesarean delivery rate observed in Class III obesity. Women with Class III obesity also had the highest median birth weight, and birth weight distribution differed significantly across groups (P<.001). High birth weight was more frequent and low birth weight was less frequent among women with Class III obesity. Diabetes differed significantly across obesity classes, whereas hypertension and deep vein thrombosis did not. No significant differences were observed across obesity classes in perineal tears, episiotomy, postpartum hemorrhage, gestational age, Apgar scores, neonatal intensive care unit admission, or birth injury. Intrauterine fetal death showed a statistically significant difference across groups, although event numbers were small.

Conclusion

Among pregnant women of advanced maternal age, increasing obesity severity, particularly Class III obesity, was associated with a higher cesarean delivery rate and higher birth weight. Most other maternal and neonatal outcomes were similar across obesity classes. These findings highlight the clinical importance of obesity class stratification in advanced maternal age pregnancies and may help inform antenatal counseling and delivery planning in high-risk obstetric populations.

## Introduction

According to the World Health Organization (WHO), obesity is defined as a body mass index (BMI) ≥30 kg/m² and is further classified into Class I (BMI 30.0-34.9 kg/m²), Class II (BMI 35.0-39.9 kg/m²), and Class III (BMI ≥40 kg/m²) [1]. The global prevalence of obesity has increased substantially over the past decades, with a marked rise among women of reproductive age [2]. Obesity has been associated with increased risk of maternal and neonatal complications during pregnancy. Complications including gestational diabetes, preterm birth, large-for-gestational-age, gestational hypertension, instrumental and caesarean birth, and surgical site infections are more likely to occur in pregnant women with obesity compared with women with a healthy weight [3].

Advanced maternal age is commonly defined as pregnancy at age 35 years or older. Pregnancy while being in this age group has been associated with increased obstetric and neonatal risks. These risks include gestational diabetes, preeclampsia, postpartum hemorrhage, maternal intensive care unit admission, stillbirth, and preterm birth [4,5]. However, both obesity and advanced maternal age, if combined, may amplify maternal and neonatal adverse risk during pregnancy.

Furthermore, the severity of obesity among advanced maternal age women, 35 years or older, is still underexplored. Prior research often evaluates obesity as a binary exposure. Fewer studies stratify obesity by classes. Even less research is done assessing obesity within advanced maternal age populations [4,6]. There is limited regional data examining whether increasing obesity is associated with maternal and neonatal complications among advanced maternal age pregnant women. This study aims to investigate the association between obesity severity (Class I, Class II, and Class III) and peripartum maternal and neonatal outcomes among advanced maternal age pregnant women (≥35 years) and evaluate whether increasing obesity class is associated with a higher likelihood of complications within this population.

## Materials and methods

Study design and setting

This study was conducted using an observational retrospective cohort design. Medical records were reviewed using the BESTCare electronic database at the Specialized Hospital for Women’s Health, Ministry of National Guard Health Affairs (MNGHA), Riyadh, Saudi Arabia, a tertiary care center providing comprehensive obstetric and neonatal services.

Study population

The study population consisted of pregnant women of advanced maternal age (≥35 years) who delivered at the hospital during the study period between 2022 and 2025.

Inclusion criteria

Women were eligible for inclusion if they were 35 years of age or older at the time of delivery, had obesity defined as BMI ≥30 kg/m², and had a recorded BMI allowing classification into obesity Class I, Class II, or Class III. Both singleton and multifetal pregnancies were eligible for inclusion.

Exclusion criteria

Patients were excluded if they were younger than 35 years, had BMI below the obesity range including underweight, normal weight, or overweight categories, or had incomplete medical records or missing key data required for the analysis.

Data collection

Data were extracted from the BESTCare electronic medical record system using a structured data collection form. Information collected included maternal demographic characteristics, obstetric history, and pregnancy outcomes. Maternal variables included maternal age, gravidity, parity, body mass index classification, and maternal comorbidities including diabetes and hypertension. Hypertension was categorized as pre-pregnancy hypertension or pregnancy-induced hypertension, with preeclampsia included under pregnancy-induced hypertension. Body mass index values recorded in the electronic medical record closest to the time of delivery were used to classify patients into obesity classes according to WHO criteria. Obstetric outcomes included mode of delivery, perineal tears, episiotomy, and postpartum hemorrhage. Neonatal variables included birth weight, gestational age at delivery, Apgar score at one and five minutes, neonatal intensive care unit admission, and intrauterine fetal death (IUFD). For multifetal pregnancies, neonatal data were collected for the first-born neonate only. Preterm birth was defined as delivery before 37 completed weeks of gestation, and birth weight was categorized as low birth weight (<2500 g), normal birth weight (2500-3999 g), and macrosomia (≥4000 g).

Statistical analysis

Statistical analysis was performed using SAS software (version 9.4, SAS Institute Inc., Cary, NC). Descriptive statistics were used to summarize maternal characteristics and pregnancy outcomes. Continuous variables were presented as median and interquartile range, while categorical variables were expressed as frequencies and percentages. Comparisons between obesity classes were performed using the chi-square test or Fisher’s exact test for categorical variables and the Kruskal-Wallis test for continuous variables. A p-value <0.05 was considered statistically significant. Analyses were performed using all available recorded data. Missing observations ranged from 0 to 37 across variables (e.g., birth weight, Apgar scores, and mode of delivery). Therefore, denominators vary slightly between analyses. Multivariable logistic regression analysis was performed as an adjusted exploratory analysis to examine factors associated with cesarean delivery, with cesarean section compared to vaginal or instrumental delivery. Maternal age, parity, diabetes status (coded as a binary variable: yes/no), and BMI class were included in the model as clinically relevant variables known to influence mode of delivery. Class I obesity was used as the reference category in the regression model.

Ethical considerations

Ethical approval for this study was obtained from the institutional review board. As the study was retrospective and used de-identified medical records, patient consent was waived and all data were handled confidentially.

## Results

A total of 1162 women were included in this study, and their demographic and clinical characteristics according to BMI class are shown in Table 1. The majority were in Class I obesity (478; 41.1%), while Class II accounted for 424 (36.5%) and Class III for 260 (22.4%). The median maternal age was 39 years (IQR 37-41). Reproductive characteristics were similar across all groups, with a median gravidity of 6 (IQR 4-7) and a median parity of 4 (IQR 3-5). There were no statistically significant differences between groups in maternal age (Kruskal-Wallis test value = 0.004, p = 0.998), gravidity (Kruskal-Wallis test value = 0.619, p = 0.734), or parity (Kruskal-Wallis test value = 0.290, p = 0.865). Regarding diabetes status, there was a statistically significant difference across obesity classes (chi-square test, χ² = 18.405, p = 0.001). Gestational diabetes was observed in 114 (23.8%) women with Class I obesity, 139 (32.8%) in Class II, and 81 (31.2%) in Class III. Pre-pregnancy diabetes occurred more frequently in Class III, 28 (10.8%), compared with Class II, 26 (6.1%), and Class I, 28 (5.9%). Out of the 1162 women, 62 had hypertension, 36 (3.1%) had pre-existing hypertension, and 26 (2.2%) had pregnancy-induced hypertension. There was no statistically significant difference in hypertension between the three groups (chi-square test, χ² = 4.467, p = 0.346). Deep vein thrombosis was rare, occurring in six women (0.5%), with no statistically significant difference between obesity classes (Fisher’s exact test, p = 0.669).

**Table 1:** Demographic and clinical characteristics according to BMI class IQR: interquartile range.

Variable	Overall (n=1162)	Class I (n=478, 41.1%)	Class II (n=424, 36.5%)	Class III (n=260, 22.4%)	p-value
Maternal age, median (IQR)	39 (37-41)	38 (37-41)	39 (37-41)	39 (37-41)	0.998
Gravidity, median (IQR)	6 (4-7)	6 (4-7)	6 (4-7)	6 (4-7)	0.734
Parity, median (IQR)	4 (3-5)	4 (3-5)	4 (3-5)	4 (3-5)	0.865
Maternal comorbidities					
Diabetes, n (%)					0.001
Gestational diabetes	334 (28.7%)	114 (23.8%)	139 (32.8%)	81 (31.2%)	
No diabetes	746 (64.2%)	336 (70.3%)	259 (61.1%)	151 (58.1%)	
Pre-pregnancy diabetes	82 (7.1%)	28 (5.9%)	26 (6.1%)	28 (10.8%)	
Hypertension, n (%)					0.346
No hypertension	1100 (94.7%)	450 (94.1%)	407 (96%)	243 (93.5%)	
Pre-pregnancy hypertension	36 (3.1%)	16 (3.3%)	8 (1.9%)	12 (4.6%)	
Pregnancy-induced hypertension	26 (2.2%)	12 (2.5%)	9 (2.1%)	5 (1.9%)	
Deep vein thrombosis, n (%)					0.669
No	1156 (99.5%)	475 (99.4%)	423 (99.8%)	258 (99.2%)	
Yes	6 (0.5%)	3 (0.6%)	1 (0.2%)	2 (0.8%)	

Maternal and neonatal outcomes according to BMI class are summarized in Table 2. A total of 1162 women were included in the analysis, of whom 478 (41.1%) had Class I obesity, 424 (36.5%) had Class II obesity, and 260 (22.4%) had Class III obesity. Mode of delivery differed significantly across BMI classes (chi-square test, χ² = 12.098, p = 0.017). The overall cesarean delivery rate was 644/1161 (55.5%). Women with Class III obesity had the highest cesarean rate at 167/259 (64.5%), compared with 228/424 (53.8%) in Class II and 249/478 (52.1%) in Class I. However, normal vaginal delivery was less frequent in Class III, 88/259 (34.0%), compared with Class I, 218/478 (45.6%), and Class II, 190/424 (44.8%). Instrumental delivery was uncommon overall, 21/1161 (1.8%), and did not show a significant difference between groups. There were no significant differences in maternal complications across BMI classes. Perineal tears occurred in 283 (24.4%) women (chi-square test, χ² = 1.831, p = 0.400), episiotomy in 62 (5.3%) (chi-square test, χ² = 0.416, p = 0.812), and postpartum hemorrhage in 110 (9.5%) (chi-square test, χ² = 2.655, p = 0.265), with similar rates across all obesity classes. The median gestational age was 38 weeks (IQR 37-39) in all groups, with no significant difference between BMI categories (Kruskal-Wallis test value = 5.211, p = 0.074). Preterm birth (<37 weeks) occurred in 228 (19.6%) pregnancies overall and was similarly distributed across BMI classes: 88 (18.4%) in Class I, 92 (21.7%) in Class II, and 48 (18.5%) in Class III (Fisher’s exact test, p = 0.440). Birth weight differed significantly between the classes (Kruskal-Wallis test value = 16.963, p < 0.001). The median birth weight was highest in Class III obesity (3130 g) compared to Class II (3002.5 g) and Class I (2997 g). High birth weight (≥4000 g) was more frequent in Class III, 10/257 (3.9%), compared with Class I, 6/472 (1.3%), and Class II, 7/422 (1.7%); however, low birth weight (<2500 g) was less common in Class III, 27/257 (10.5%), compared with Class I, 89/472 (18.9%), and Class II, 72/422 (17.1%) (chi-square test, χ² = 14.100, p = 0.007). Apgar scores at one and five minutes were similar across the BMI classes. The median Apgar score at one minute was 9 (IQR 8-9) and at five minutes was 9 (IQR 9-9), with no significant differences (Kruskal-Wallis test value = 4.154, p = 0.125; Kruskal-Wallis test value = 0.431, p = 0.806, respectively). The proportion of neonates with Apgar score <7 at one minute was 99/1126 (8.8%) overall and did not differ significantly between the classes (chi-square test, χ² = 0.731, p = 0.694). Similarly, Apgar score <7 at five minutes occurred in 38/1125 (3.4%) neonates without significant variation (chi-square test, χ² = 1.265, p = 0.531). NICU admission was required in 134 (11.5%) neonates and showed no significant difference across the classes (chi-square test, χ² = 1.335, p = 0.513). Birth injury was rare, occurring in three (0.3%) neonates, and was comparable between groups (Fisher’s exact test, p = 0.243). However, IUFD occurred in 22 (1.9%) pregnancies and differed significantly across BMI classes (chi-square test, χ² = 6.080, p = 0.048).

**Table 2:** Maternal and neonatal outcomes according to BMI class Percentages and summary statistics are based on available data for each variable. IQR: interquartile range; n: number; NICU: neonatal intensive care unit.

Outcomes	Overall (n=1162)	Class I (n=478, 41.1%)	Class II (n=424, 36.5%)	Class III (n=260, 22.4%)	p-value
Maternal outcomes					
Mode of delivery					0.017
Cesarean delivery	644 (55.5%)	249 (52.1%)	228 (53.8%)	167 (64.5%)	
Instrumental delivery	21 (1.8%)	11 (2.3%)	6 (1.4%)	4 (1.5%)	
Normal vaginal delivery	496 (42.7%)	218 (45.6%)	190 (44.8%)	88 (34%)	
Perineal tear, n (%)					0.400
No	878 (75.6%)	351 (73.6%)	327 (77.1%)	200 (76.9%)	
Yes	283 (24.4%)	126 (26.4%)	97 (22.9%)	60 (23.1%)	
Episiotomy, n (%)					0.812
No	1100 (94.7%)	454 (95%)	399 (94.1%)	247 (95%)	
Yes	62 (5.3%)	24 (5%)	25 (5.9%)	13 (5%)	
Postpartum haemorrhage, n (%)					0.265
No	1051 (90.5%)	434 (90.8%)	389 (91.7%)	228 (88%)	
Yes	110 (9.5%)	44 (9.2%)	35 (8.3%)	31 (12%)	
Neonatal outcomes					
Gestational age, median (IQR)	38 (37-39)	38 (37-39)	38 (37-39)	38 (37-39)	0.074
Gestational age					0.44
Pre-term: < 37 weeks	228 (19.6%)	88 (18.4%)	92 (21.7%)	48 (18.5%)	
Term: 37 – 41 weeks	931 (80.1%)	388 (81.2%)	332 (78.3%)	211 (81.2%)	
Post-term: ≥ 42 weeks	3 (0.3%)	2 (0.4%)	0 (0%)	1 (0.4%)	
Birth weight, median (IQR)	3040 (2680-3340)	2997 (2630-3270)	3002.5 (2650-3350)	3130 (2800-3460)	<0.001
Birth weight					0.007
<2500 g	188 (16.3%)	89 (18.9%)	72 (17.1%)	27 (10.5%)	
≥4000 g	23 (2%)	6 (1.3%)	7 (1.7%)	10 (3.9%)	
2500-3999 g	940 (81.7%)	377 (79.9%)	343 (81.3%)	220 (85.6%)	
Apgar score at one minute, median (IQR)	9 (8-9)	9 (8-9)	9 (8-9)	9 (8-9)	0.125
Apgar score at five minutes, median (IQR)	9 (9-9)	9 (9-9)	9 (9-9)	9 (9-9)	0.806
Apgar score categories					
<7 at one min	99 (8.8%)	44 (9.5%)	32 (7.9%)	23 (9%)	0.694
7-10 at one min	1027 (91.2%)	420 (90.5%)	375 (92.1%)	232 (91%)	
<7 at five min	38 (3.4%)	19 (4.1%)	12 (3%)	7 (2.7%)	0.531
7-10 at five min	1087 (96.6%)	445 (95.9%)	394 (97%)	248 (97.3%)	
NICU admission, n (%)					0.513
No	1028 (88.5%)	429 (89.7%)	372 (87.7%)	227 (87.3%)	
Yes	134 (11.5%)	49 (10.3%)	52 (12.3%)	33 (12.7%)	
Birth injury, n (%)					0.243
No	1159 (99.7%)	475 (99.4%)	424 (100%)	260 (100%)	
Yes	3 (0.3%)	3 (0.6%)	0 (0%)	0 (0%)	
Intrauterine fetal death, n (%)					0.048
No	1140 (98.1%)	464 (97.1%)	417 (98.3%)	259 (99.6%)	
Yes	22 (1.9%)	14 (2.9%)	7 (1.7%)	1 (0.4%)	

Maternal and neonatal characteristics according to the mode of delivery are shown in Table 3. Of the 1161 deliveries with available mode-of-delivery data, more than half, 644/1161 (55.5%), were delivered by cesarean section, while 496/1161 (42.7%) had normal vaginal delivery. Instrumental delivery was uncommon, occurring in 21/1161 (1.8%) cases. The overall median maternal age was 39 years (IQR 37-41). Gravidity and parity showed a statistically significant association with mode of delivery (Kruskal-Wallis test values = 18.439 and 23.301, respectively; p < 0.001). Women with higher median gravidity of 6 (IQR 4-8) and parity of 4 (IQR 3-5) were more likely to have normal vaginal delivery. In contrast, women who underwent instrumental delivery had lower median gravidity of 4 (IQR 2-5) and parity of 2 (IQR 1-3). Maternal BMI also demonstrated a statistically significant association with the mode of delivery (chi-square test, χ² = 12.098, p = 0.017). Among women who underwent cesarean section, 249/644 (38.7%) were from Class I obesity, and 167/644 (25.9%) were from Class III obesity. In the normal vaginal delivery group, 218/496 (44.0%) were from Class I obesity, and Class III accounted for 88/496 (17.7%). Infant birth weight was within the normal range (2500-3999 g) in most cases, 940/1150 (81.7%), and showed a statistically significant association with mode of delivery (Fisher’s exact test, p = 0.004). Among the cesarean section delivery group, low birth weight (<2500 g) occurred in 123/637 (19.3%) of cases, while the majority had normal birth weight, 498/637 (78.2%). In the normal vaginal delivery group, most neonates had normal birth weight, 423/492 (86.0%), while only 63/492 (12.8%) had low birth weight.

**Table 3:** Maternal and neonatal characteristics according to the mode of delivery Percentages and summary statistics are based on available data for each variable. One case had missing mode of delivery data. BMI: body mass index; IQR: interquartile range.

Variables	Overall (n=1161)	Cesarean Delivery (n=644, 55.5%)	Instrumental Delivery (n=21, 1.8%)	Normal Vaginal Delivery (n=496, 42.7%)	p-value
Maternal age, median (IQR)	39 (37-41)	39 (37-41)	38 (36-39)	38 (37-41)	0.058
BMI class, n (%)					0.017
Obesity Class I	478 (41.2%)	249 (38.7%)	11 (52.4%)	218 (44%)	
Obesity Class II	424 (36.5%)	228 (35.4%)	6 (28.6%)	190 (38.3%)	
Obesity Class III	259 (22.3%)	167 (25.9%)	4 (19%)	88 (17.7%)	
Gravidity, median (IQR)	6 (4-7)	6 (4-7)	4 (2-5)	6 (4-8)	<0.001
Parity, median (IQR)	4 (3-5)	4 (2-5)	2 (1-3)	4 (3-5)	<0.001
Birth weight, n (%)					0.004
<2500 g	187 (16.3%)	123 (19.3%)	1 (4.8%)	63 (12.8%)	
≥4000 g	23 (2%)	16 (2.5%)	1 (4.8%)	6 (1.2%)	
2500-3999 g	940 (81.7%)	498 (78.2%)	19 (90.5%)	423 (86%)	

Adjusted exploratory logistic regression results for factors associated with cesarean delivery are shown in Table 4. A multivariable logistic regression analysis was performed as an adjusted exploratory analysis of factors associated with cesarean delivery. Using obesity Class I as the reference category, women with Class III obesity had significantly higher odds of cesarean delivery (odds ratio (OR) 1.62, 95% confidence interval (CI) 1.19-2.22, z = 3.028, p = 0.002), indicating an approximately 62% increase in the odds of cesarean section relative to Class I obesity. In contrast, Class II obesity showed no significant association with cesarean delivery (OR 1.04, 95% CI 0.80-1.36, z = 0.315, p = 0.752). Diabetes was also associated with an increased likelihood of cesarean delivery in the adjusted model. Women with diabetes had 31% higher odds of cesarean section relative to non-diabetic women (OR 1.31, 95% CI 1.02-1.68, z = 2.099, p = 0.035). Maternal age showed no significant association with cesarean delivery after adjustment for other variables (OR 1.04, 95% CI 0.99-1.08, z = 1.492, p = 0.135). Higher parity was associated with lower odds of cesarean section in the adjusted model. Every extra birth was associated with a 6% reduction in the odds of cesarean section (OR 0.94, 95% CI 0.88-0.99, z = -1.991, p = 0.046). In the adjusted model, Class III obesity, diabetes, and parity showed significant associations with cesarean delivery.

**Table 4:** Multivariable logistic regression for predictors of cesarean delivery BMI: body mass index; OR: odds ratio; CI: confidence interval. Diabetes was included as a binary (yes/no) variable.

Variable	OR	95% CI	p-value
BMI class: Class I obesity	Reference	-	-
BMI class: Class II obesity	1.043	0.801-1.359	0.752
BMI class: Class III obesity	1.624	1.187-2.224	0.002
Diabetes	1.307	1.018-1.679	0.035
Maternal age	1.036	0.989-1.084	0.135
Parity	0.938	0.881-0.999	0.046

## Discussion

The main goal of this study was to investigate the association between maternal obesity class and peripartum maternal and neonatal outcomes among women of advanced maternal age who were admitted to a specialized women’s hospital in Riyadh, Saudi Arabia. The findings demonstrate that the severity of maternal obesity, particularly Class III obesity, was associated with delivery outcomes, including cesarean delivery. The main findings of this study were the higher cesarean delivery rate and higher birth weight observed among women with Class III obesity.

Maternal obesity is now regarded as an important issue in global health, especially for women of reproductive age. Despite the variations in the body mass index classification (BMI), the demographic characteristics of the population, including parity, were comparable across all obesity groups. Therefore, the differences in outcomes may be related to the level of obesity rather than demographic characteristics. Previous studies have shown that obesity is highly prevalent in pregnant women and is associated with adverse outcomes [7].

One of the most significant findings in this study was the relationship between obesity severity and mode of delivery. The results indicated that the rate of cesarean delivery was higher among women with Class III obesity compared to those with Class I obesity, whereas the rate of vaginal delivery was lower among women with Class III obesity. Fallatah et al. reported a significant increase in the rate of cesarean delivery among obese pregnant women in Saudi Arabia, particularly among those with higher BMI classes [8]. Neal et al. also indicated that obesity severity was independently associated with the rate of cesarean delivery among obese pregnant women in Australia [6].

In the adjusted analysis, maternal diabetes was also associated with cesarean delivery. This finding should be interpreted cautiously, as obesity severity and diabetes are clinically interrelated and may partially overlap in their contribution to adverse obstetric outcomes. Previous studies have shown that pregnancies complicated by maternal diabetes are associated with higher rates of cesarean section and increased risk of adverse birth outcomes [9].

Previous studies have shown inconsistent findings regarding the relationship between parity and cesarean section. However, our study showed that higher parity was associated with a reduced likelihood of cesarean section in the adjusted model, with each additional birth reducing the likelihood of cesarean section by approximately 6%.

In contrast to the observed differences in delivery method, our study did not show significant differences in several maternal complications among obesity groups, including perineal tears, episiotomy, and postpartum hemorrhage. Although some studies have indicated higher rates of postpartum complications in obese women, Fallatah et al. reported a significant association between obesity and postpartum hemorrhage, as well as vaginal tears [8].

A significant difference was noted in neonatal birth weight, with newborns of mothers with Class III obesity having a higher median birth weight than those of mothers with Class I and Class II obesity. This finding supports earlier evidence linking maternal obesity with increased fetal growth.

However, there were no significant differences in other neonatal outcomes, including Apgar scores at one and five minutes, NICU admission, and birth injuries. This is consistent with the literature suggesting that advanced maternal age may not significantly affect neonatal outcomes during the peripartum period when appropriate antenatal care is provided. A systematic review by Rodrigues et al. showed inconsistent results regarding the effect of advanced maternal age on neonatal morbidity [10].

This study has several strengths. First, it was conducted on a considerable number of women of advanced maternal age, enabling comparison across all classes of obesity. Second, this study was carried out in a specialized women’s health hospital, providing a relatively standardized clinical setting. Additionally, an adjusted exploratory analysis of factors associated with cesarean delivery was performed.

This study has a few limitations, which should be acknowledged. First, the study was retrospective, and this design is associated with several methodological challenges. Second, BMI classification was based on values recorded near delivery since pre-pregnancy BMI measurements were not consistently available. These near-delivery measurements may have been influenced by gestational weight gain. Small numbers for some outcomes, such as IUFD and instrumental delivery, also limited interpretation. Furthermore, the study was conducted in a single center, which may limit generalizability. Several potentially relevant clinical and non-clinical confounding variables, including socioeconomic factors, weight gain, and lifestyle variables, were not included in the study. Multifetal gestation was not recorded as a separate study variable and therefore could not be evaluated independently, which may have influenced some maternal and neonatal outcome estimates. Because obesity severity and diabetes are clinically interrelated, the adjusted regression findings should be interpreted cautiously. A prospective multicenter study would help provide broader understanding.

## Conclusions

In conclusion, greater severity of maternal obesity, particularly Class III obesity, was associated with a higher rate of cesarean delivery among advanced maternal age women. In addition, newborns of mothers with Class III obesity had higher birth weights compared with those of mothers with lower obesity classes. However, no significant differences were observed in maternal complications and most neonatal outcomes across obesity classes. These findings highlight the clinical importance of obesity class stratification in advanced maternal age pregnancies and may help inform antenatal counseling and delivery planning in this high-risk population.
